# Functional Exploration of the Polysaccharide Lyase Family PL6

**DOI:** 10.1371/journal.pone.0159415

**Published:** 2016-07-20

**Authors:** Sophie Mathieu, Bernard Henrissat, Flavien Labre, Gudmund Skjåk-Bræk, William Helbert

**Affiliations:** 1 CERMAV, CNRS and Grenoble Alpes Université, BP53, 38000, Grenoble, Cedex 9, France; 2 Centre National de la Recherche Scientifique (CNRS), UMR7257, Université Aix-Marseille, 13288, Marseille, France; 3 INRA, USC 1408 AFMB, 13288, Marseille, France; 4 Department of Biotechnology, Norwegian University of Science and Technology, NTNU Sem Sælands vei 6–8, 7491, Trondheim, Norway; Weizmann Institute of Science, ISRAEL

## Abstract

Alginate, the main cell-wall polysaccharide of brown algae, is composed of two residues: mannuronic acid (M-residues) and, its C5-epimer, guluronic acid (G-residues). Alginate lyases define a class of enzymes that cleave the glycosidic bond of alginate by β-elimination. They are classified according to their ability to recognize the distribution of M- and G-residues and are named M-, G- or MG-lyases. In the CAZy database, alginate lyases have been grouped by sequence similarity into seven distinct polysaccharide lyase families. The polysaccharide lyase family PL6 is subdivided into three subfamilies. Subfamily PL6_1 includes three biochemically characterized enzymes (two alginate lyases and one dermatan sulfatase lyase). No characterized enzymes have been described in the two other subfamilies (PL6_2 and PL6_3). To improve the prediction of polysaccharide-lyase activity in the PL6 family, we re-examined the classification of the PL6 family and biochemically characterized a set of enzymes reflecting the diversity of the protein sequences. Our results show that subfamily PL6_1 includes two dermatan sulfates lyases and several alginate lyases that have various substrate specificities and modes of action. In contrast, subfamilies PL6_2 and PL6_3 were found to contain only endo-poly-MG-lyases.

## Introduction

Alginate is the main structural cell-wall component of brown algae (Phaeophyceae) and is also produced by a limited number of Gram-negative bacteria. It is a linear copolymer made of two building units, β-D-mannuronic acid (M) and its C5 epimer α-L-guluronic acid (G). The two residues are linked by β(1,4) linkages and are distributed along the polysaccharide chain as consecutive M-residues (M-blocks), G-residues (G-blocks) or alternating M- and G-residues (MG-blocks). The texturizing and gelling properties of alginate in the presence of divalent cations (e.g. Ca^++^, Mg^++^)—of particular interest for industrial applications—are correlated with the composition and distribution of the M- and G-residues [1]

Alginate lyases define class of enzymes that cleave the alginate glycosidic bonds *via* a β-elimination mechanism leading to the formation of an unsaturated residue, 4-deoxy-*erythro*-hex-4-enopyrasyluronate, at the non-reducing end of the products. Alginate lyases are classified according to their substrate specificities and refer to the block structures encountered in alginate. For example, poly-β-D-mannuronate lyase (M-lyase, EC 4.2.2.3,) and poly-α-L-guluronate lyase (G-lyase, EC 4.2.2.11) cleave the glycosidic bond between M-M or G-G moieties, respectively. M-G blocks can be cleaved by M-G-lyase or G-M-lyase according to the recognition properties of the enzyme [2]

Alginate lyases have been also classified by sequence similarity and are found in seven families of polysaccharide lyases (PL) in the carbohydrate-active enzyme database (CAZy, http://www.cazy.org/; [3]). To date, five PL families include characterized enzymes that are active on alginate (families PL5, PL7, PL15, PL17 and PL18); however different substrate specificities have been recorded: e.g. both G-lyases and MG-lyases are included in the PL7 family [4, 5]. Two other PL families are polyspecific meaning that the alginate lyases are grouped with enzymes inactive on alginate, but active on chondroitin B (PL6; [6]) or on β-D-polyglucuronic acid (PL14; [7]). In an attempt to improve the prediction of substrate specificity based on sequence data, a subfamily classification of PLs has been proposed following an approach similar to that applied to polyspecific glycoside hydrolase families GH13 [8], GH5 [9], GH30 [10] and GH43 [11]. Based on available biochemical data, most of the proposed GH subfamilies are monospecific and differences within a subfamily generally involve the endo- or exolytic mode of action of the enzymes [12].

Family PL6 is a polyspecific family that contains 177 proteins listed in the CAZy database (June 2016), all of which are of bacterial origin and among which only three representatives (i.e. about 2%) have been biochemically characterized. Family PL6 is divided into three subfamilies, but only subfamily PL6_1 has characterized members, namely chrondroitinase B lyase [13] and a specific poly-MG lyase [14]. The third characterized enzyme is an alginate lyase that has not been classified in any subfamily, because of the divergence of its protein sequence compared with the other members of the PL6 family [15]. Thus, subfamily PL6_1 appears to be polyspecific and there are no functionally characterized members in the two other subfamilies.

To improve the functional prediction of family PL6 members based on sequence data, we reexamined the subfamilies within PL6. We also biochemically characterized a series of enzymes spanning the diversity of proteins from family PL6. Elucidation of the function of PL6 enzymes belonging to any of the three subfamilies or as yet unclassified provided insight on the functional evolution of the family and helped determine the common catalytic mechanism that prevails in this family.

## Materials and Methods

### Bioinformatics of polysaccharide lyases belonging to family PL6

Protein accessions of 137 non-fragmentary family PL6 members were extracted from the CAZy database (December 2015) and used to retrieve the corresponding amino-acid sequences from the NCBI database. After addition of the sequence from *Nonlabens ulvanivorans* ORF Nonul_2381 (WP_036584323.1), the amino-acid sequences were trimmed to isolate the PL6 catalytic domain based on the three-dimensional structure of chondroitinase B from *Pedobacter heparinus* (PDB code 1DBO). The resulting 138 sequences were aligned using MUSCLE [16]. The alignment was used to compute a distance matrix based on maximum-likelihood distances [17] with BLOSUM62 substitution parameters [18]. The resulting distance matrix was then used to construct a phylogenetic tree that was divided into subfamilies using Secator [19].

### Cloning of alginate lyase genes from family PL6

Fifteen genes were selected to cover the sequence diversity of the PL6 family. The choice of the targets was also determined by the commercial availability of DNA material or by the selection of easily culturable aerobic bacterial strains. Genomic DNA and bacterial strains were obtained from the DSMZ-German Collection of Microorganisms and Cell Cultures. The gene names, their accession number and the template (purified genomic DNA or bacterial clones) used for cloning are listed in Table 1.

**Table 1:** Sources of the genetic material. The genes were cloned directly on the strains or on purified DNA. The gene *sven0074* was synthesized and optimized for expression in *E*. *col*i.

Strains	Reference	Template used for cloning	Genes	Accession number
*Alteromonas macleodii*	DSM6062	genomic DNA	*mase*04135	AFS36376
			*mase*04180	AFS36385
*Cellulophaga lytica*	DSM7489	bacteria	*celly*0294	ADY28129
*Nonlabens ulvanivorans*	DSM**22727**	bacteria	*Nonul2381*	WP_036584323.1
*Pedobacter heparinus*	DSM2366	bacteria	*phep*3223	ACU05418
*Pedobacter saltans*	DSM12145	genomic DNA	*pedsa*3807	ADY54336
			*pedsa*0631	ADY51207
			*pedsa*0632	ADY51208
			*pedsa*3628	ADY54157
*Pseudoalteromonas atlantica T6c*	DSM6840	bacteria	*patl*3640,	ABG42142
			*patl*3659	ABG42161
*Rhodothermus marinus*	DSM4252	genomic DNA	*rmar*1165,	ACY48055
			*rmar*1386	ACY48275
*Saccharophagus degradans*	DSM17024	genomic DNA	*sde*0034	ABD79298
*Streptomyces venezuelae*	DSM40230	Synthetic gene	*sven*0074	CCA53362

The presence of signal peptides and their site of cleavage were identified using SignalP [20]. The primers designed for the amplification of the targeted genes without the signal peptide are listed in Table 2. The primers contained *Nco*I/*Xho*I, *BamH*I*/EcoR*I, *BamH*I*/Xho*I or *Nhe*I*/Hind*III pairs of restriction enzyme sites; all compatible for cloning in the pET28a expression plasmid (Novagen, USA). Because no expression was obtained when *mase*04180 was cloned into pET28a, this insert was also cloned into pET32a (Novagen, USA) using the same restriction enzymes (*Nco*I/*Xho*I).

**Table 2:** Cloning primers. The primers were designed, as much as possible, to be used with the same restriction enzymes for parallel cloning. Position of the His-tag in the protein, expected size and predicted pI of the proteins are also indicated.

Restriction sites	Genes	Primers (5’ → 3’)	position of His_6_Tag	MW (kDa)	pI
*NcoI/XhoI*	*rmar1165*-F	GTG***CCATGG***CCGTCCGCTACGTG	C-ter	63.0	6.2
	*rmar1165*-R	GT***CTCGAG***GGGCCGAGCGATG			
*NcoI/XhoI*	*mase04135*-F	GAC***CCATGG***GAAAACAATATACCGTTAGCTCACCAG	C-ter	81.3	5.9
	*mase04135*-R	G***CTCGAG***CCTCTTGTTCGCTTTCGTGG			
*NcoI/XhoI*	*celly0294*-F	CAT***CCATGG***GAAATACCGTAGCTACTTTAC	C-ter	84.5	7.3
	*celly0294*-R	CC***CTCGAG***GTTATTACTAATTAATCCTAAATTTTTGCC			
*NcoI/XhoI*	*pedsa0631*-F	CCT***CCATGG***GAAACATAACCGTTTCCACTCC	C-ter	84.9	8.6
	*pedsa0631*-R	CC***CTCGAG***ATTGATTTTCGCTCCTAATGCC			
*NcoI/XhoI*	*pedsa0632*-F	CAG***CCATGG***GATCCGAAAAAATAACAGAC	C-ter	48.8	7.6
	*pedsa0632*-R	C***CTCGAG***AAAGTTCATTTTAACATTATTCCATTCC			
*NcoI/XhoI*	*pedsa3628*-F	CAA***CCATGG***GAAATATTCAAGTAAAAAATGCTTCAGAAC	C-ter	51.1	9.0
	*pedsa3628*-R	CC***CTCGAG***GATTGACCACTTAACACC			
*NcoI/XhoI*	*mase04180*-F	GTT***CCATGG***GTGCTACAAACCAGCCTG	C-ter	103.2	4.1
	*mase04180*-R	G***CTCGAG***GTTACTCTGAGCCGGTG			
*NcoI/XhoI*	*pedsa3807*-F	CGA***CCATGG***GAAAGCATATTCTTGTTGC	C-ter	56.6	8.5
	*pedsa3807*-R	C***CTCGAG***GTTATTTCTGTCTCTTGCC			
*BamHI/EcoRI*	*patl3640*-F	CAAA***CCATGG***GATTAGTTGAAAACATCAAACAGTTC	N-ter	79.9	5.6
	*patl3640*-R	CT***CTCGAG***GTCGTAAGTCACATTTGAGC			
*BamHI/EcoRI*	*patl3659*-F	AAAA***CCATGG***ATACTTTAGTCAAAACGCCTGAA	N-ter	81.5	6.4
	*patl3659*-R	AAA***AAGCTT***TAGCGTTTTGTTTCCGCTGATAA			
*BamHI/XhoI*	*sde0034*-F	GTT***GGATCC***GCAGACCCAGTTTCTGTTG	N-ter	81.0	4.8
	*sde0034*-R	GG***CTCGAG***TTAAGGCGCACTTGG			
*NheI/HindIII*	*rmar1386*-F	CCT***GCTAGC***ACGGTTGTGCTGAACACTTC	N-ter	107.0	4.8
	*rmar1386*-R	C***AAGCTT***TTACCTGATCAGGGCAAGTTTGTAAGC			
*NheI/HindIII*	*phep3223*-F	CTT***GCTAGC***TTTGCAGGTACAATTACTGTTTC	N-ter	49.7	8.5
	*phep3223*-R	C***AAGCTT***CTATTTCAGGTCTGCAGTATGC			

Solutions of genomic DNA in TE buffer (10 mM Tris pH 7.5, 1 mM EDTA) provided by the DSMZ collection were diluted 1:10 in distilled water and 0.5 μL aliquots were used as templates. When cloning was conducted directly on a bacterial strain, isolated colonies on agar petri dishes were picked and suspended in 10 μL distilled water but only 0.5 μL was used as template. Amplification of the gene by PCR was carried out on a Master Cycler gradient machine (Eppendorf). Amplification products were purified on agarose gel with Wizard® SV Gel and PCR clean-up system kit (Promega). DNA fragments were digested with their respective restriction enzymes (FastDigest–Thermo Fischer Scientific) at least 1 h at 37°C and purified again with SureClean kit (Bioline). The plasmid vectors were also digested with the corresponding pair of restriction enzymes and purified on agarose gel as described above. Ligation was performed using a TAKARA ligation kit (Clontech), 30 min at 16°C, in a final volume that allow the ligation of 10 to 50 ng of linearized vector and an amount of purified insert corresponding to a ratio insert: vector of about 3. A maximum of 7 μL of ligation product was transformed into competent *E*. *coli* Top10 cells and the newly constructed plasmid was verified by sequencing (MWG Operon, Germany).

Amplification of the gene *sven*0074 failed, presumably because of the very high GC content of the *Streptomyces venezuelae* genome. Therefore, the gene was synthesized and optimized for expression in *E*. *coli* (MWG Operon, Germany). It was designed with flanking *Nco*I and *Xho*I restriction sites at the 5' and 3' ends, respectively, for cloning into the pET28a vector according to the above protocol.

### Heterologous expression and purification of recombinant PL6 lyases

*E*. *coli* BL21(DE3) cells harboring the recombinant expression plasmids were cultured on Luria Bertani (LB) medium supplemented with 50 μg/mL kanamycin (for the pET28a plasmid) or 100 μg/mL ampicillin (for the pET32a plasmid) until the OD_600nm_ reached 0.6 in a shaking incubator at 180 rpm and 37°C. After the addition of 0.2 mM isopropyl-β-D-thiogalactopyranoside to induce heterologous expression, the temperature was cooled to 20°C and maintained overnight.

Cultures were stopped by centrifugation at 6000 x*g* for 5 min. The bacterial pellet was suspended in buffer A (20 mM Tris pH 8, 500 mM NaCl) before lysis with a French press. Insoluble fractions were removed by centrifugation at 40,000 x*g* for 30 min at 4°C. The proteins were purified by affinity using a nickel agarose affinity resin (Ni-NTA resin, Qiagen) loaded on poly-prep® chromatography columns (Bio-Rad 731–1550). The resin was equilibrated with buffer A and the His_6_-tagged recombinant enzymes were eluted with buffer A containing increasing amounts of imidazole (20 mM, 50 mM, 150 mM, 300 mM and 500 mM). The purity of the fractions was estimated by 10% SDS-PAGE analysis.

### Alginate substrates

High-molecular-weight mannuronan (poly-M) (fraction of guluronic acid F_G_ = 0, [η] = 940 mL/g) was produced by an *algG*^*-*^ mutant strain of *Pseudomonas fluorescence* according to [21]. Alginate with a strictly alternating poly-MG sequence (F_G_ = 0.46 and F_GG_ = 0), was prepared by incubating poly-M with recombinant mannuronan-C-5 epimerase AlgE4 from *Azotobacter vinelandii* expressed in *Hansenula polymorpha* [22]. Poly-G (F_G_ = 0.95, DP_n_ = 20) was prepared from *Laminaria hyperborea* according to [23].

#### Degradation kinetics

Enzymatic assays were carried out by incubating of 1.2 mL of alginate (0.2% w/v in 50 mM Tris-HCl pH 8, 1 mM CaCl_2_) with 10 μL of purified enzyme at 25°C. The protein concentration was adapted to each targeted enzyme and ranged from 1 to 5 μM. The production of reducing ends was measured using the ferricyanide method. Aliquots (40 μL) were transferred to a 200 μL ferricyanide solution (1.5 g/L K_3_[Fe(CN)_6_], 24 g Na_2_CO_3_, 1 mL 5 M NaOH, qsp 1 L) which stopped the enzymatic reaction. The solution was heated to 100°C for 10 min (described below) and, after cooling to room temperature, the absorbance of 200 μL of sample was measured at 415 nm with a microplate reader (Model 680 Bio-Rad). For chromatography experiments, the enzymatic reaction was stopped by heating the sample to 100°C for 10 min.

### Gel permeation chromatography

#### Analytical

After filtration (0.2 μm), samples were eluted on 0.1 M NaCl pre-equilibrated Superdex S200 10/300 and Superdex peptide 10/300 (GE Healthcare) columns mounted in series and connected to a high-performance liquid chromatography (HPLC) Ultimate 3000 system (Thermo Fischer). The injection volume was 20 μL and the elution was performed at 0.4 mL/min in the same eluent. Oligosaccharides were detected by differential refractometry (Iota 2 differential refractive index detector, Precision Instruments) and UV spectrometry at 235 nm (Thermo Fischer).

#### Semi-preparative chromatography

End-products were purified on a semi-preparative size-exclusion chromatography system which consisted of a Knauer pump (pump model 100), a HW40 Toyopearl column (120 x 16 mm; Tosoh Corporation), a refractive index detector (Iota 2, Precision Instruments) and a fraction collector (Foxy R1) mounted in series. Elution was conducted at a flow rate of 0.4 mL/min at room temperature using 100 mM (NH_4_)_2_CO_3_ as the eluent. The fractions containing pure oligosaccharides (mono-, di- or tetrasaccharides: DP1, DP2, DP4, respectively) were collected and freeze-dried.

### ^1^H NMR

Samples were exchanged twice with D_2_O and were transferred to a 5 mm NMR tube. ^1^H NMR spectra were recorded at 323 K using an Advance III 400 MHz spectrometer (Bruker). Chemical shifts are expressed in ppm in reference to water. The HOD signal was not suppressed.

### Molecular weight determination

Samples (100 μL) filtered on 0.22 μm membrane were injected in Shodex OH pack SB 802.5 HQ and Shodex OH pack SB 802 HQ columns mounted in series. Elution was performed with 0.1 M NaNO_3_ at a flow rate of 0.5 mL/min (Shimadzu LC-20AD pump) at 25°C. This HPLC system was coupled to a Wyatt Optilab Rex refractive index detector, used as a mass-sensitive detector, working at 633 nm at 25°C. Laser light scattering measurements were performed at 633 nm with a mini Dawn TREOS system (Wyatt technology). The intensity of scattered light was measured at three different angles, 45°, 90° and 135°. Chromatographic data were collected and processed using the Astra software (Wyatt technology). The calculated d*n*/d*c* (refractive index increment) was 0.115 mL/g. Toluene was to calibrate the detector.

## Results

### Phylogenetic tree of the PL6 family

**Fig 1:**
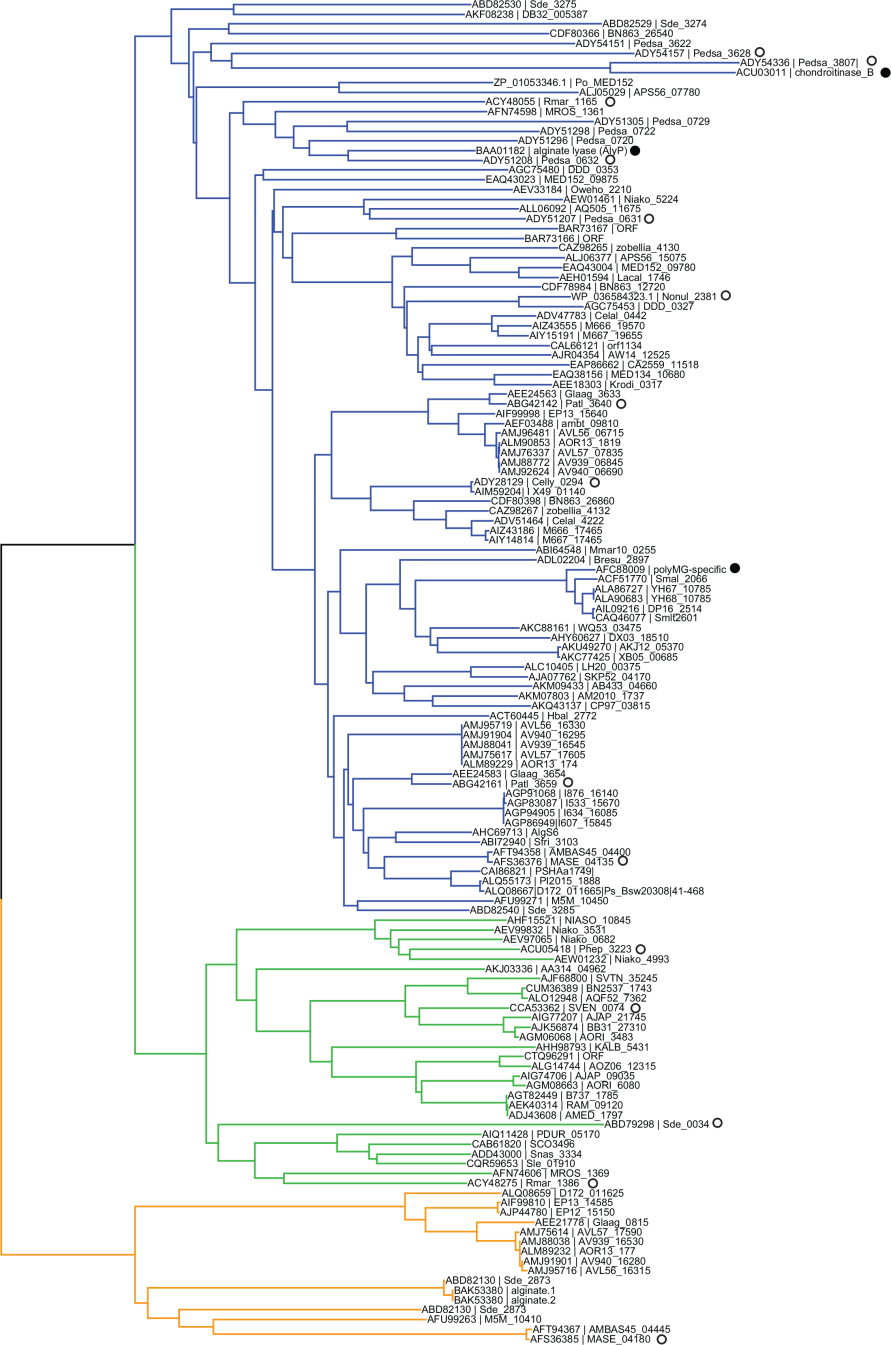
Phylogenetic tree of the PL6 family. Characterized enzymes (●) reported in the literature and enzymes analyzed in this work (○) are indicated. The branches of the tree that correspond to subfamilies 1, 2 and 3 are colored in blue, green and orange, respectively.

The phylogenetic tree of the sequences corresponding to the catalytic domain of 138 PL6 family members (Fig 1) shows a clear subdivision into three clades that coincide with subfamilies PL6_1, PL6_2 and PL6_3, as indicated in the CAZy database. Interestingly, all three characterized PL6 enzymes belong to subfamily PL6_1. Therefore the activity that prevails in the two other subfamilies cannot be predicted with accuracy (Lombard et al. 2010). We thus used the phylogenetic tree as a guide to select a panel of enzymes for expression and characterization, and focused on the uncharacterized subfamilies PL6_2 and PL6_3 and on regions of subfamily PL6_1 that were distant from the previously characterized enzymes.

### Cloning, heterologous expression and one-step purification of PL6 enzymes

Fifteen target genes spread throughout the phylogenetic tree were selected to span the diversity of the PL6 protein sequences. All 15 genes were successfully amplified by PCR except the *sven*0074 gene whose sequence was optimized for expression in *E*. *coli*, and then successfully cloned in pET28a. The cloned gene *mase*04180 was not expressed in pET28a and was therefore cloned in pET32a in which there was satisfactory expression of the soluble protein coupled to thioredoxin.

All proteins fused to a His_6_ tag were successfully overexpressed in *E*. *coli* BL21(DE3) grown at 20°C overnight. The proteins were purified by affinity on a Ni^2+^-bound chelating sepharose matrix. Overexpression of soluble proteins was analyzed by SDS-PAGE and observed molecular weights ranged from 47.0 kDa to 107 kDa, showing expected sizes.

### Determination of the preferred substrates

**Fig 2:**
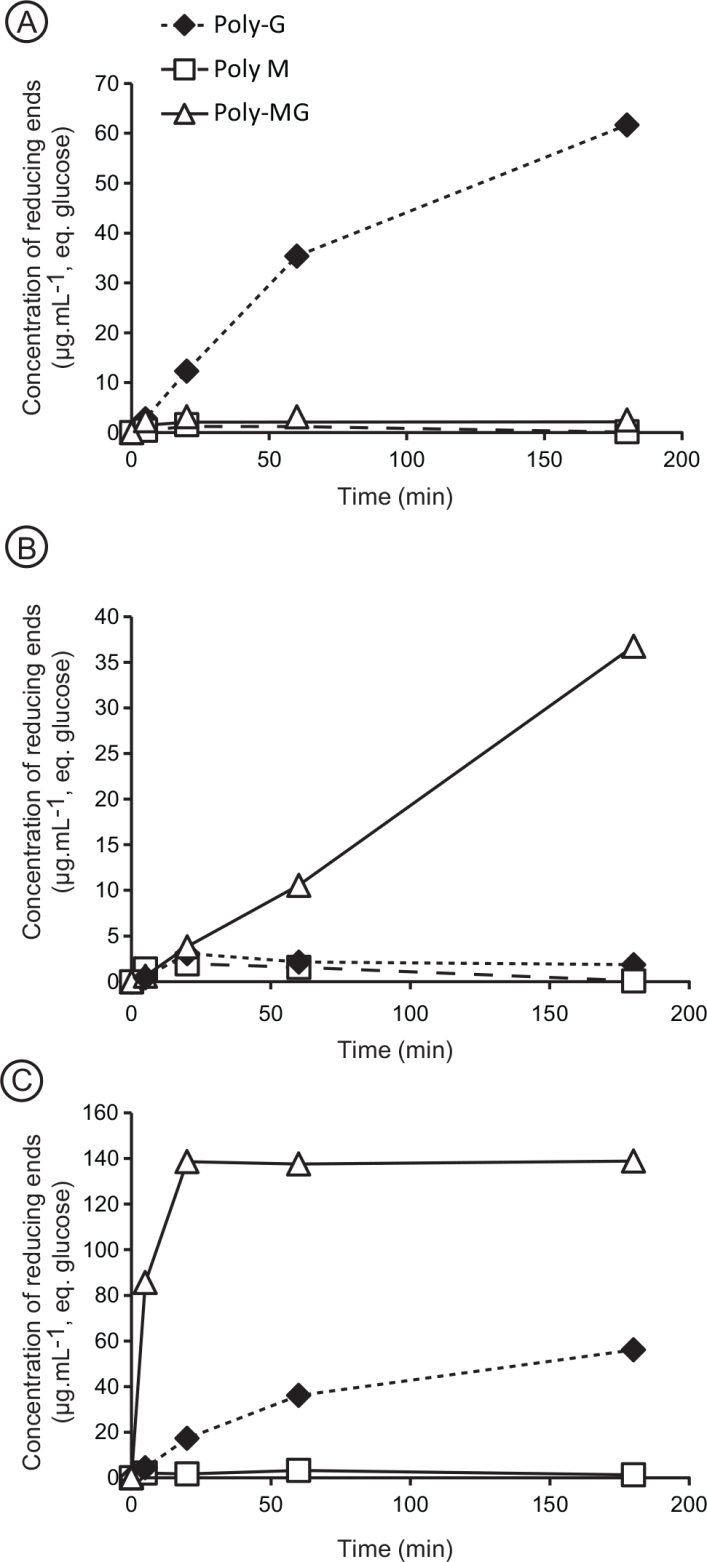
Degradation kinetics of alginate. Enzymatic degradation of poly-M, poly-G and poly-MG was monitored by measuring the production of reducing ends. The substrates were incubated with (**A**) Patl3640, (**B**) Sven0074, and (**C**) Pedsa0632.

The purified proteins were incubated with poly-M, poly-G and poly-MG alginates. Degradation kinetics of alginate were followed by monitoring the formation of reducing ends using the ferricyanide method. Incubation of poly-G with Patl3640 led to the production of an increasing amount of reducing ends with time (Fig 2A), but poly-M and poly-MG were not degraded despite addition of enzyme in excess. Similarly, the alginate lyase Sven0074 cleaved the glycosidic bond of poly-MG (Fig 2B), but was inefficient on poly-G. The substrate specificity of these two enzymes (Patl3640, Sven0074) was therefore unambiguously identified. However, when an enzyme was able to digest two different substrates, we considered that the preferred substrate was the one that showed the highest degradation velocity. For instance, alginate lyase Pedsa0632 degraded poly-MG and poly-G (Fig 2C). The number of cleavages increased more rapidly with poly-MG than with poly-G. Therefore, poly-MG was considered the preferred substrate and poly-G the secondary substrate.

Enzyme specificity was determined for all selected enzymes and data are reported in Table 3. Degradation kinetics revealed that the proteins classified in subfamilies PL6_2 and PL6_3 acted only on poly-MG, thus their specific substrate. In subfamily PL6_1, results were more contrasted. Most of the alginate lyases were specific to poly-MG, but were also able to digest poly-G as a secondary substrate. Two alginate lyases (Patl3640, Pedsa0631) of subfamily PL6_1 were not active on poly-MG but were active only on poly-G. The enzyme Pedsa3807, which is highly homologous to chondroitinase B lyase (syn. dermatan sulfate lyase) [13] and also placed in subfamily PL6_1, was not active on alginate, but cleaved dermatan sulfate as expected.

**Table 3:** Substrate specificity and mode of action. The table summarize the substrate specificities, end-products and mode of action (endo-, exo-) of the polysaccharide lyases investigated.

Genes	Strains	Preferred substrate	End-products	Mode of action	Other substrate	End-products	Mode of action
**Sub-family 1**							
Pedsa3628	*Pedobacter saltans*	Poly MG	ΔM / ΔMGM	Endo	-	-	-
Rmar1165	*Rhodothermus marinus*	Poly MG	ΔM / ΔMGM	Endo	Poly G	Δ	Exo
Nonul2381	*Nonlabens ulvanivorans*	Poly MG	ΔM / ΔMGM	Endo	Poly G	Δ	Exo
Patl3659	*Pseudoalteromonas atlantica T6c*	Poly MG	ΔM / ΔMGM	Endo	Poly G	Δ	Exo
Celly0294	*Cellulophaga lytica*	Poly MG	ΔM / ΔMGM	Endo	Poly G	Δ	Exo
Mase04135	*Alteromonas macleodii*	Poly MG	ΔM / ΔMGM	Endo	Poly G	Δ	Exo
Pedsa0632	*Pedobacter saltans*	Poly MG	ΔM / ΔMGM	Endo	Poly G	Δ/ΔGG(G)	Endo
Patl3640	*Pseudoalteromonas atlantica T6c*	Poly G	Δ	Exo	-	-	-
Pedsa0631	*Pedobacter saltans*	Poly G	Δ	Exo	-	-	-
Pedsa3807	*Pedobacter saltans*	CS / DS	Active with Mn^2+^	Endo			
**Sub-family 2**							
Phep3223	*Pedobacter heparinus*	Poly MG	ΔM / ΔMGM	Endo	-	-	-
Rmar1386	*Rhodothermus marinus*	Poly MG	ΔM / ΔMGM	Endo	-	-	-
Sven0074	*Streptomyces venezuelae*	Poly MG	ΔM / ΔMGM	Endo	-	-	-
Sde0034	*Saccharophagus degradans 2–40*	Poly MG	ΔM / ΔMGM	Endo	-	-	-
**Sub-family 3**							
Mase04180	*Alteromonas macleodii*	Poly MG	ΔM / ΔMGM	Endo	-	-	-

### Analysis of the mode of action

**Fig 3:**
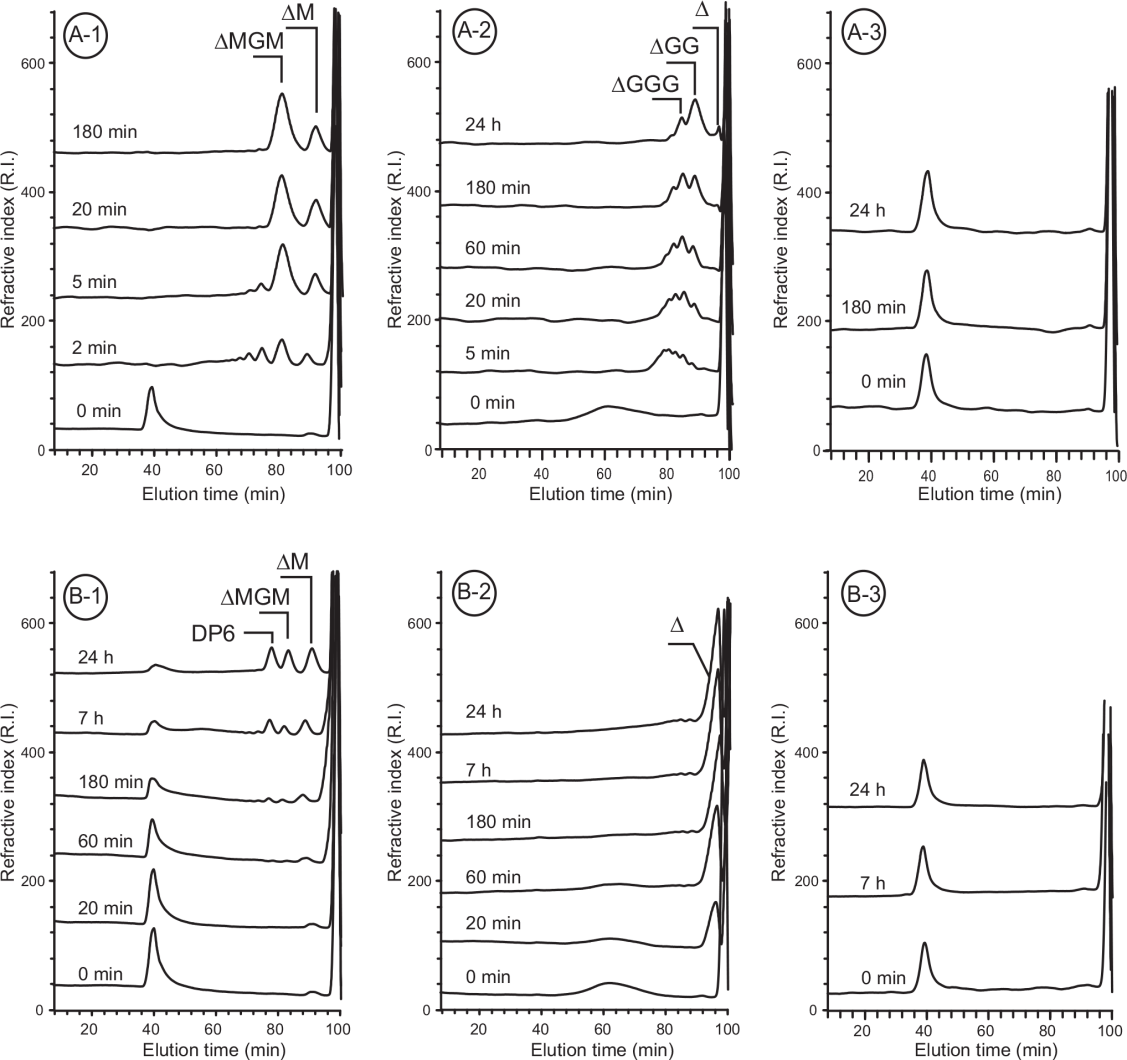
Size-exclusion chromatography monitoring the degradation of alginate substrates. Kinetic chromatograms correspond to the degradation of poly-MG (**A-1**, **B-1**), poly-G (**A-2**, **B-2**) and poly-M (**A-3**, **B-3**) by Pedsa0632 (**A**) and Mase04135 (**B**). The end-products: Δ, ΔM, ΔMGM, are indicated on the chromatograms.

Size-exclusion chromatography was used to determine the endo- or exolytic mode of action of the alginate lyases (Fig 3). Endo-acting enzymes cleave the polymeric substrate into a range of oligosaccharides whose sizes decrease as the degradation proceeds. Conversely, exo-acting enzymes lead to the production of a single end-product. Two enzymes of subfamily PL6_1 illustrate our results (Fig 3A and 3B). Incubation of poly-MG (Fig 3A-1) and poly-G (Fig 3A-2) with Pedsa0632 led to the production of a range of oligosaccharides, forming a range of oligosaccharides from high molecular weights to DP4 and DP2. This pattern of degradation is consistent with an endolytic mode of action for Pedsa0632 on poly-MG and poly-G alginates. According to the degradation kinetics given in Fig 2B, alginate lyase Mase04135 also has an endolytic mode of action on poly-MG (Fig 3B-1). However, the degradation of poly-G led to a single end-product (DP1) suggesting an exo- mode of action for this substrate (Fig 3B-2).

**Fig 4:**
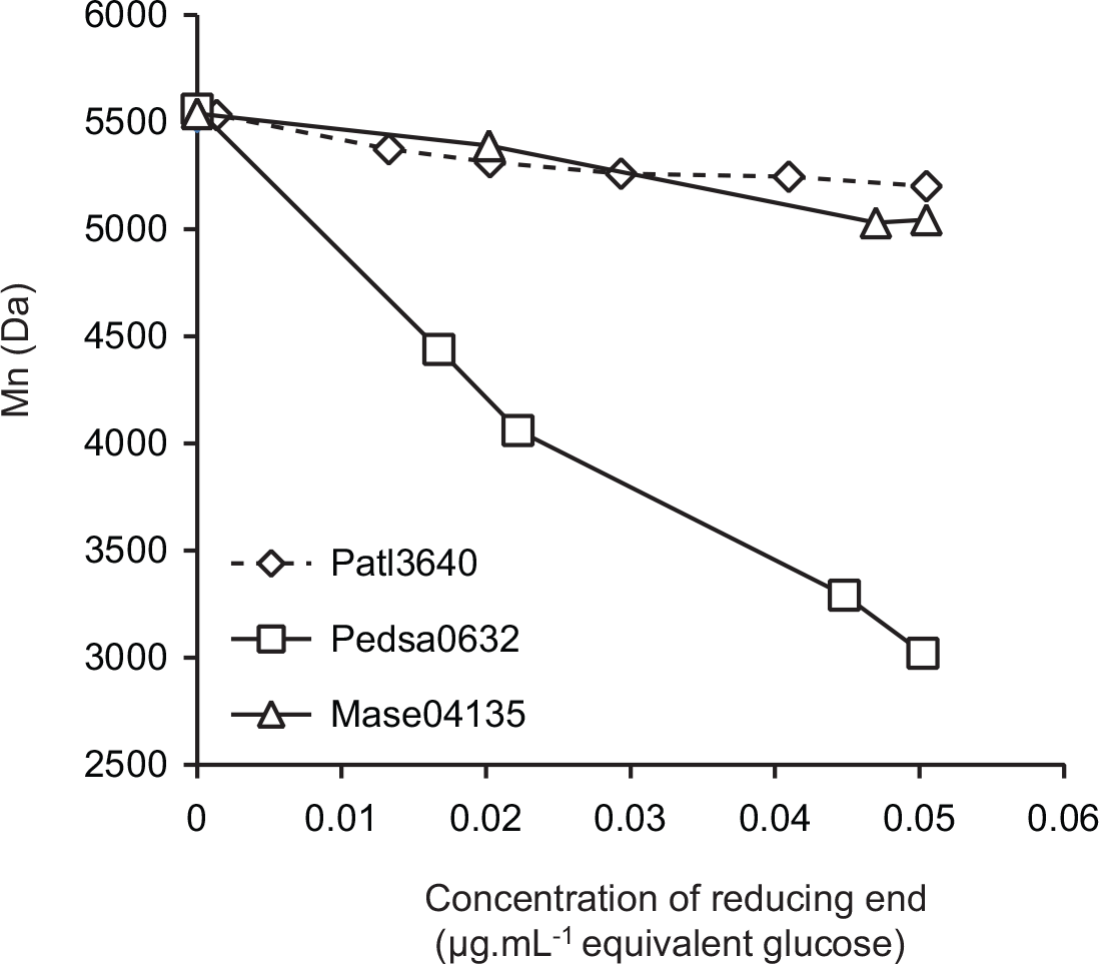
Kinetics of poly-G depolymerization. The variation in molecular mass (Mn) is expressed as a function of the concentration of reducing ends formed during alginate degradation.

Modes of action were confirmed by analysis of the variation in molecular mass of poly-G measured by LC-laser light scattering as a function of the number of cleavages (Fig 4). The rapid decrease in the molecular mass of poly-G after incubation with Pedsa0632 confirmed the endolytic character of this alginate lyase. This observation contrasted with the variation in molecular mass of poly-G incubated with Mase04135. In the latter case, the molecular mass decreased only slightly, confirming the exolytic mode of action for Mase04135. The results also exclude a potential endo-processive mode of action which leads to a rapid loss of molecular weight caused by of the endolytic mode of action event though only one main end-product is generated due to the processive action. The mode of action of all the enzymes was determined using size-exclusion chromatography and, for exo-enzymes, confirmed by LC-MALLS analysis. The results summarized in Table 3 show that the degradation of poly-MG always occurs according to an endolytic mode of action. All enzymes active on poly-G except for Pedsa0632 showed an exolytic mode of action on this substrate (Table 3).

### Structure of the end-products of poly-MG lyases

**Fig 5:**
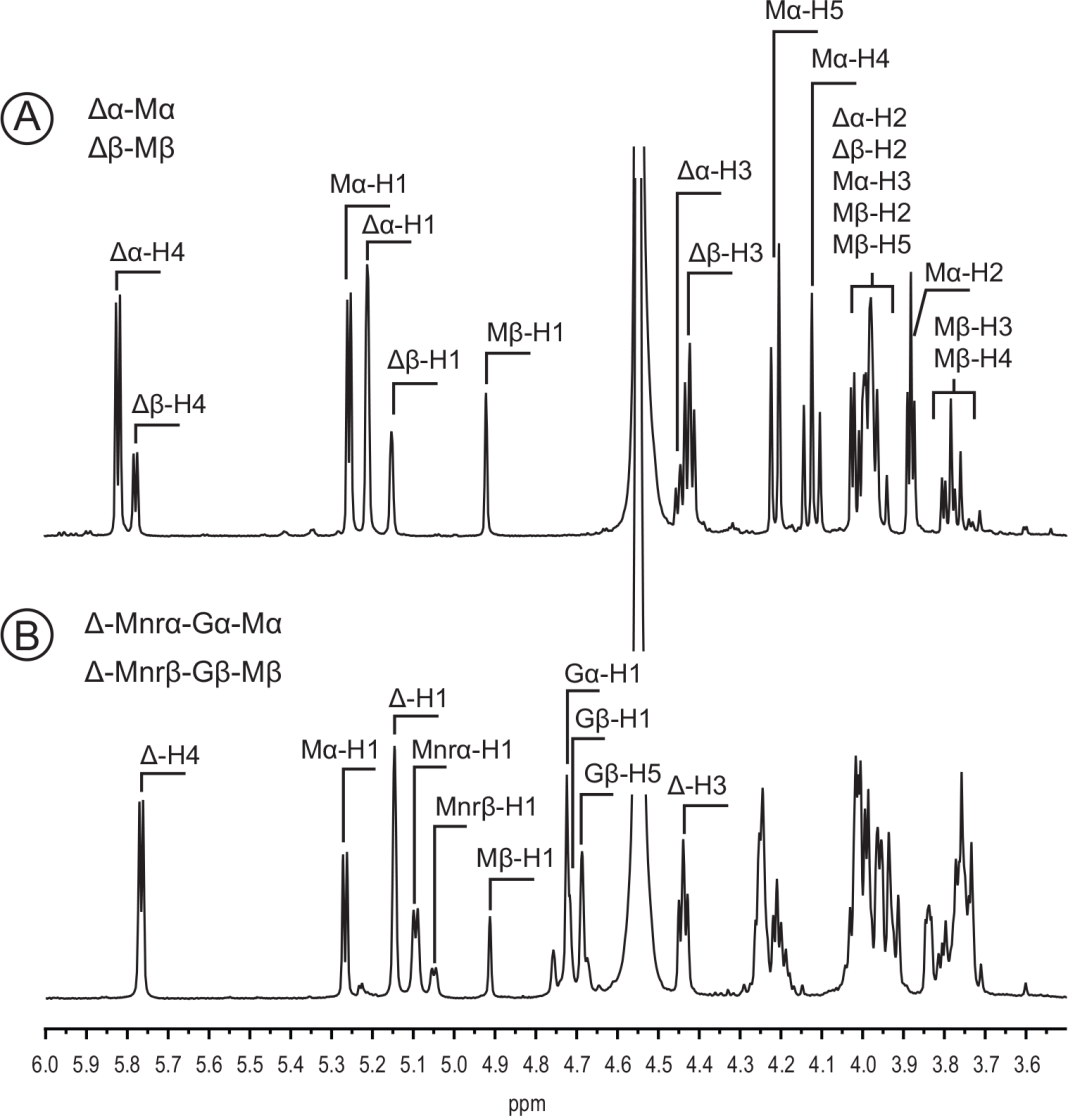
^1^H NMR spectra of the end-products of upon incubation with poly-MG lyase. A and B are the spectra of the disaccharide and tetrasaccharide end-products, respectively. The proton signal were attributed.

End-products obtained after complete degradation of poly-MG were purified by semi-preparative chromatography and were analyzed by NMR. ^1^H NMR spectra presented in Fig 5A show all the chemical shifts of a disaccharide (DP2) composed of M-residues at the reducing end and unsaturated 4-deoxy-*erythro*-hex-4-enopyranyluronate at the non-reducing end. The ^1^H NMR spectra are the superimposed spectra of two different DP2 that lie on the α/β-anomeric configuration of the mannuronic residue. The chemical shifts of the H4 protons of the unsaturated residues (Δ-H4) were measured at 5.78 ppm (Δα-H4) when the M-residue adopted an α-anomer configuration or at 5.82 ppm (Δβ-H4) for the β-anomer configuration. The ring protons were ascribed straightforwardly using 2D NMR analyses (COSY, HSQC, HMBC, not shown). In particular, the anomeric protons of the unsaturated residues were observed at 5.21 ppm (Δα-H1) and 5.14 ppm (Δβ-H1). The signals of the α- and β-anomeric protons of at the reducing end had proton chemical shifts of 4.94 ppm (M β- H1) and 5.27 ppm (Mα-H1), which correspond to carbon chemical shifts of 93.2 ppm (Mα-C1) and 93.7 ppm (M β-C1), respectively. Starting from the anomic protons, most of the ring protons were also ascribed using 2D NMR analyses. The *J*^*3*^_H1-H2_ coupling of the α-anomer of 3.4 Hz is in agreement with an axial-axial configuration of the protons. The proton H5 measured at 4.23 ppm (Mα-H5) presented a *J*^*3*^_H4-H5_ coupling constant of 7.7 Hz confirming the axial-axial configuration of the protons. The observed chemical shifts were clearly different from those reported for the disaccharide ΔG [24]. Furthermore, the coupling constants *J*^*3*^_H1-H2_ and *J*^*3*^_H4-H5_ were characteristic of mannuronic residues, demonstrating the Δ-Mα/β structure of this end-product.

The second end-product was digested into Δ-M, the unique end-product after prolonged incubation with Pedsa0632 and Sven0074 alginate lyases, suggesting that it is a tetrasaccharide (DP4) with a Δ-M-G-Mα/β structure. NMR analyses of this oligosaccharide confirmed that the reducing end was an M-residue (Mα-H1: 5.27 ppm; M β-H1: 4.91). The residue linked to the reducing M-residue is a G-residue with anomeric protons measured at 4.72 ppm (Gα-H1) and 4.71 ppm (Gβ-H1). The G-H5 signal was observed at 4.69 ppm. The *J*^*3*^_H1-H2_ and *J*^*3*^_H4-H5_ constants of the G-residue were weak and in agreement with an equatorial-equatorial configuration of the protons in the α-L-guluronic acid residues. The end-products obtained after digestion of poly-MG were all analyzed by NMR and all had Δ-M and Δ-M-G-M structures. Therefore, the alginate lyases cleave glycosidic bonds between the M-G moieties, but not between G-M moieties. These results demonstrate that the active site of the poly-MG lyases in the PL6 family accommodate the M-residues in subsite -1 and the G residue in subsite +1.

## Discussion

A phylogenetic analysis of protein sequences showed that the PL6 family is divided into three subfamilies, for each of which several representatives were biochemically characterized. In particular, we carried out the first analysis of representatives from subfamilies 2 and 3 and showed that they all have specific endo-poly-MG lyase activity. Most of the enzymes grouped in subfamily 1 had alginate activities, but differed in their substrate specificities and their mode of action. Alginate lyases have either endo-poly-MG/exo-poly-G lyase activity, endo-poly-MG/endo-poly-G lyase activity (Pedsa0632) or strict exo-poly-G lyase activity (Patl3640, Pedsa0631). The previously characterized *Stenotrophomas maltophilia* KJ-2 [14] also falls in subfamily 1 and the reported endo-poly-MG mode of action is in agreement with our observations. The characterized but unclassified in CAZy *Pseudomonas sp*. OS-ALG-9 alginate lyase [15] also grouped with subfamily 1, but published enzymatic assays do not allow identification of its substrate specificity. The *Pedobacter saltans* chondroitinase B lyase Pedsa3807 was also classified in subfamily 1 increasing the functional diversity inside the subfamily 1.

The aim of partitioning the PL6 family into subfamilies was to improve protein sequence annotation and functional prediction. Biochemical characterization of PL6 enzymes revealed that members of subfamilies 2 and 3 have similar strict endo-poly-MG lyase activity. Therefore, sub-classification does not seem to be correlated with functional differences of PL6 enzymes. The characterized polysaccharide lyases belonging to subfamily 1 showed diverse substrate specificities and modes of action. There was no correlation between the degradation properties of the enzymes and its subfamily based on protein sequence. For example, the strict exo-G lyases (Patl3640, Pedsa0631) did not cluster together but were found on distant branches within subfamily 1. In addition to alginate lyases, subfamily 1 also contained chondroitinase B lyase found near the root of subfamily 1. Therefore, current partitioning of PL6 subfamily 1 does not lead to any straightforward prediction of substrate specificity or mode of action.

The subfamily classification in GH13 [8], GH5 [9], GH30 [10] and GH43 [11] is based on a high number of non-redundant sequences producing reliable trees with a robust, discriminating subfamily classification in agreement with substrate diversity. The PL6 tree is based on a relatively small set of sequences and, although it highlights three subfamilies, it does not yet reflect substrate diversity. In particular, a larger set of sequences may reveal that subfamily 1, which includes enzymes with various specificities and modes of action, actually contains several subfamilies.

The three subfamilies encompassed alginate lyases and most of the unclassified enzymes were also alginate lyases. Therefore, the common ancestor of this family was likely an alginate lyase. The diversity of enzyme activity encountered in PL6 subfamilies suggests an evolutionary pathway from strict endo-poly-MG lyases to exo-poly-G lyase *via* an intermediate situation with enzymes having both endo-poly-MG/exo-poly-G lyase activities. The end-products of poly-MG lyases demonstrate that the catalytic subsite +1 always harbors a G residue. Therefore, the shift from MG to GG substrate moieties required remodeling subsite -1 to accommodate a G-residue instead of an M-residue. This remodeling did not require modification of the catalytic machinery because the reactive G-residue (located in +1) remains unchanged.

The chondroitinase B lyase activity observed with Pedsa3807 *Pedobacter saltans* lyase was expected because of its high sequence homology with *Flavobacterium heparinum* chondroitinase B lyase [13]. Dermatan sulfate is a galactosaminoglycan composed of a repeating disaccharide unit of 1,4- β-D-*N*-acetyl-galactosamine sulfated at position 4 and 1,3-α-L-iduronic acid. Stereochemistry of the residues, modalities of their linkages as well as their decoration with sulfate and *N*-acetyl groups are quite different from those observed in alginates and indicate that protein evolution from alginate lyase to chondroitinase B lyase was possible only after a major remodeling of the active site.

Crystal structure of *Flavobacterium heparinum* chondroitinase B lyase and its complex with several oligosaccharides has shed light on the β-elimination mechanism in the PL6 family [25, 26]. As proposed by Gacesa [27], the first step of the β-elimination consists in neutralizing the carboxyl group which is ensured by a Ca^2+^ in the PL6 family. Then, abstraction of the proton at C5 by a conserved lysine leads to the formation of an enolate anion intermediate. Finally, electron transfer from the carboxyl group to form a double bond between C4 and C5 and stepwise or concomitant protonation of the β(1,4) linkage by a conserved arginine lead to the cleavage of the glycosidic bond. This mechanism requires that the configuration of the carboxyl group and the proton at C5 of the uronic residue in position +1 be conserved throughout the PL6 family. The iduronic acid of dermatan and the guluronic acid of alginate belong to the L-series, meaning that they have the same asymmetry at the C5 carbon. In addition, the pyranose ring of L-guluronic acid preferentially adopts a ^1^C_4_ conformation, whereas the L-iduronic acid ring shows more flexibility and adopts ^1^C_4_, ^4^C_1_ and ^2^S_0_ conformations. Therefore, the spatial arrangement of the carboxyl group and the proton at C5 are common to L-iduronic and L-guluronic acids and, as expected, can use the same catalytic machinery for the first steps of the β-elimination.

The protons at the C4 and C5 positions are on the same side (*syn* configuration) of the L-guluronic acid ring. In the case of L-iduronic acid, the C4 and C5 proton sets have opposing positions (*anti* configuration). The last step of the β-elimination, which consists of the protonation of the oxygen in the glycosidic bond by a conserved arginine, is probably independent of the *syn* or *anti* configuration. Nevertheless, the distance between the catalytic amino acid and the glycosidic bond must be conserved. In a reflection of the diversity of substrate specificities and modes of action encountered in subfamily PL6_1, the *syn* or *anti* configuration of protons does not appear to be conserved either in this subfamily.

In conclusion, biochemical characterization of a set of enzymes selected based on their protein sequence allowed an exploration of the diversity of enzyme activities encountered in the PL6 family. This investigation strategy of a PL family aims to facilitate functional prediction and help select targets for further crystallographic studies. In PL6, no alginate lyase structures have been solved crystallographically. Therefore, we identified potential targets that will help understand the functional evolution of the PL6 family.

## References

[1] Draget KI, Smidsrød O, Skjak-Braek G. In: De Baets S, Vandamme E, Steinbüchel A (eds) Biopolymers 6; polysaccharides II: polysaccharides from Eucaryotes. 2002 Wiley, Weinheim

[2] Garron ML, Cygler M. (2010) Structural and mechanistic classification of uronic acid-containing polysaccharide lyases. Glycobiology 2010; 20: 1547–1573 10.1093/glycob/cwq122 20805221

[3] Cantarel BL, Coutinho PM, Rancurel C, Bernard T, Lombard V, Henrissat B. The Carbohydrate-Active EnZymes database (CAZy): an expert resource for Glycogenomics. Nucleic Acids Res. 2009; 37: D233–238 10.1093/nar/gkn663 18838391 PMC2686590

[4] Yamasaki M, Moriwaki S, Miyake O, Hashimoto W, Murata K, Mikami B. Structure and function of a hypothetical *Pseudomonas aeruginosa* protein PA1167 classified into family PL-7: a novel alginate lyase with a beta-sandwich fold. J Biol Chem. 2004; 279: 31863–31872. 15136569 10.1074/jbc.M402466200

[5] Osawa T, Matsubara Y, Muramatsu T, Kimura M, Kakuta Y. Crystal structure of the alginate (poly alpha-l-guluronate) lyase from *Corynebacterium* sp. at 1.2 A resolution. J Mol Biol. 2005; 345: 1111–1118 15644208 10.1016/j.jmb.2004.10.081

[6] Huang W, Matte A, Li Y, Kim YS, Linhardt RJ, Su H et al Crystal structure of chondroitinase B from *Flavobacterium heparinum* and its complex with a disaccharide product at 1.7 A resolution. J Mol Biol. 1999; 294: 1257–1269 10600383 10.1006/jmbi.1999.3292

[7] Ogura K, Yamasaki M, Yamada T, Mikami B, Hashimoto W, Murata K (2009) Crystal structure of family 14 polysaccharide lyase with pH-dependent modes of action. J Biol Chem 284: 35572–35579 10.1074/jbc.M109.068056 19846561 PMC2790987

[8] Stam MR, Danchin EG, Rancurel C, Coutinho PM, Henrissat B. Dividing the large glycoside hydrolase family 13 into subfamilies: towards improved functional annotations of α-amylase-related proteins. Protein Eng Des Sel. 2006; 19: 555–562 17085431 10.1093/protein/gzl044

[9] Aspeborg H, Coutinho PM, Wang Y, Brumer H, Henrissat B. Evolution, substrate specificity and subfamily classification of glycoside hydrolase family 5 (GH5). BMC Evol Biol. 2012; 12: 186 10.1186/1471-2148-12-186 22992189 PMC3526467

[10] St John FJ, González JM, Pozharski E (2010) Consolidation of glycosyl hydrolase family 30: a dual domain 4/7 hydrolase family consisting of two structurally distinct groups. FEBS Lett 584: 4435–4441 10.1016/j.febslet.2010.09.051 20932833

[11] Mewis K, Lenfant N, Lombard V, Henrissat B. Dividing the large glycoside hydrolase family 43 into subfamilies: a motivation for detailed enzyme characterization. Appl Environ Microbio. 2016; 82: 1686–92 10.1128/AEM.03453-15 PMC4784025 26729713

[12] Lombard V, Bernard T, Rancurel C, Brumer H, Coutinho PM, Henrissat B. A hierarchical classification of polysaccharide lyases for glycogenomics. Biochem J. 2010; 432: 437–44 10.1042/BJ20101185 20925655

[13] Tkalec AL, Fink D, Blain F, Zhang-Sun GY, Laliberte M, Bennett DC et al Isolation and expression in *Escherichia coli* of *cslA* and *cslB*, genes coding for the chondroitin sulfate-degrading enzymes chondroitinase AC and chondroitinase B, respectively, from *Flavobacterium heparinum*. Appl Environ Microbiol. 2000; 66: 29–35 10618199 10.1128/aem.66.1.29-35.2000 PMC91781

[14] Lee SI, Choi SH, Lee EY, Kim HS. Molecular cloning, purification, and characterization of a novel polyMG-specific alginate lyase responsible for alginate MG block degradation in *Stenotrophomas maltophilia* KJ-2. Appl Environ Microbiol. 2012; 95: 1643–1653 10.1007/s00253-012-4266-y 22805784

[15] Kraiwattanapong J, Ooi T, Kinoshita S. Cloning and sequence analysis of the gene (alyII) coding for an alginate lyase of Pseudomonas sp. OS-ALG-9. Biosci Biotech Biochem. 1997; 61: 1853– 10.1271/bbb.61.1853 9404064

[16] Edgar RC. MUSCLE: multiple sequence alignment with high accuracy and high throughput. Nucleic Acids Res. 2004; 32: 1792–1797 15034147 10.1093/nar/gkh340 PMC390337

[17] Guindon S, Gascuel O. A simple, fast, and accurate algorithm to estimate large phylogenies by maximum likelihood. Systematic Biol. 2003; 52: 696–704 10.1080/10635150390235520 14530136

[18] Henikoff JG. Amino acid substitution matrices from protein blocks. Proc Natl Acad Sci. USA 1992; 89: 10915–10919 1438297 10.1073/pnas.89.22.10915 PMC50453

[19] Wicker N, Perrin GR, Thierry JC, Poch O. Secator: A program for inferring protein subfamilies from phylogenetic trees. Mol Biol Evol. 2001; 18: 1435–1441 11470834 10.1093/oxfordjournals.molbev.a003929

[20] Petersen TN, Brunak S, von Heijne G, Nielsen H. SignalP 4.0: discriminating signal peptides from transmembrane regions. Nature Methods 2011; 8:785–786 10.1038/nmeth.1701 21959131

[21] Gimmestad M, Sletta H; Ertesvag H; Bakkevig K, Jain S, Suh S et al The *Pseudomonas fluorescens* AlgG protein, but not its mannuronan C-5-epimerase activity, is needed for alginate polymer formation. J Bacteriol. 2003; 185: 3515–3523. 12775688 10.1128/JB.185.12.3515-3523.2003 PMC156231

[22] Aarstad OA, Tøndervik A, Sletta H, Skjåk-Bræk G. Alginate sequencing. An analysis of block distribution in alginates using specific alginate degrading enzymes. Biomacromol. 2012; 13: 106–116. 10.1021/bm2013026 22148348

[23] Haug A, Larsen B, and Smidsrød O. Studies of the sequence of uronic acid residues in alginic acid. Acta Chem. Scand. 1967; 21: 691–704B

[24] Heyraud A, Gey C, Leonard C, Rochas C, Girond S, Kloareg B. NMR spectroscopy analysis of oligoguluronates and oligomannuronates prepared by acid or enzymatic hydrolysis of homopolymeric blocks of alginic acid. Application to the determination of the substrate specificity of *Haliotis tuberculata* alginate lyase. Carbohydr Res. 1996; 289: 11–23 8805773 10.1016/0008-6215(96)00060-2

[25] Huang WJ, Matte A, Li YG, Kim YS, Linhardt RJ, Su HS, et al Crystal structure of chondroitinase B from *Flavobacterium heparinum* and its complex with a disaccharide product at 1.7 Å resolution. J Mol Biol. 1999; 294: 1257–1269 10600383 10.1006/jmbi.1999.3292

[26] Michel G, Pojasek K, Li YG, Sulea T, Linhardt RJ, Raman R et al The structure of chondroitin B lyase complexed with glycosaminoglycan oligosaccharides unravels a calcium-dependent catalytic machinery. J Biol Chem. 2004; 279: 32882–32896 15155751 10.1074/jbc.M403421200 PMC4135467

[27] Gacesa P. Alginate-modifying enzymes. A proposed unified mechanism of action for the lyases and epimerases. FEBS 1987; 212: 199–202

